# Phosphorylation of collapsin response mediator protein-2 disrupts neuronal maturation in a model of adult neurogenesis: Implications for neurodegenerative disorders

**DOI:** 10.1186/1750-1326-6-67

**Published:** 2011-09-24

**Authors:** Leslie Crews, Rebecca Ruf, Christina Patrick, Wilmar Dumaop, Margarita Trejo-Morales, Cristian L Achim, Edward Rockenstein, Eliezer Masliah

**Affiliations:** 1 Department of Neurosciences; University of California, San Diego; 9500 Gilman Drive, La Jolla, CA 92093-0624, USA; 2 Department of Pathology; University of California, San Diego; 9500 Gilman Drive, La Jolla, CA 92093, USA; 3 Department of Psychiatry; University of California, San Diego; 9500 Gilman Drive, La Jolla, CA 92093, USA

**Keywords:** neurogenesis, HIV, encephalitis, CRMP2, dpysl2, CDK5, microtubules, neurite outgrowth

## Abstract

**Background:**

Recent studies suggest that the pathogenic process in neurodegenerative disorders may disrupt mature neuronal circuitries and neurogenesis in the adult brain. Abnormal activation of CDK5 is associated with neurodegenerative disorders, and recently a critical role for CDK5 in adult neurogenesis has been identified. We have developed an *in vitro *model of abnormal CDK5 activation during adult hippocampal neurogenesis, and here we used this model to investigate aberrantly phosphorylated downstream targets of CDK5.

**Results:**

Abnormal CDK5 activation in an *in vitro *model of adult neurogenesis results in hyperphosphorylation of collapsin-response mediator protein-2 (CRMP2) and impaired neurite outgrowth. Inhibition of CDK5, or expression of a non-phosphorylatable (S522A) CRMP2 construct reduced CRMP2 hyperphosphorylation, and reversed neurite outgrowth deficits. CRMP2 plays a role in microtubule dynamics; therefore we examined the integrity of microtubules in this model using biochemical and electron microscopy techniques. We found that microtubule organization was disrupted under conditions of CDK5 activation. Finally, to study the relevance of these findings to neurogenesis in neurodegenerative conditions associated with HIV infection, we performed immunochemical analyses of the brains of patients with HIV and transgenic mice expressing HIV-gp120 protein. CDK5-mediated CRMP2 phosphorylation was significantly increased in the hippocampus of patients with HIV encephalitis and in gp120 transgenic mice, and this effect was rescued by genetic down-modulation of CDK5 in the mouse model.

**Conclusions:**

These results reveal a functional mechanism involving microtubule destabilization through which abnormal CDK5 activation and CRMP2 hyperphosphorylation might contribute to defective neurogenesis in neurodegenerative disorders such as HIV encephalitis.

## Background

During aging and in the progression of neurodegenerative conditions such as Alzheimer's disease (AD) and HIV-associated neurocognitive disorders, synaptic plasticity and neuronal integrity are disturbed [1-3]. Although the precise mechanisms leading to neurodegeneration in these conditions remain unclear, some common signaling factors have been identified that contribute to the pathogenesis of multiple neurodegenerative processes. One important signaling molecule that may represent a common denominator in several neurodegenerative disorders is cyclin-dependent kinase-5 (CDK5). Previous studies have revealed that dysregulation of CDK5 and its activators p35 and p25 contribute to the abnormal accumulation of hyperphosphorylated CDK5 substrates and eventual mature neuronal cell death in AD, HIV-associated neuroinflammatory conditions such as HIV encephalitis (HIVE), and prion-related disorders such as scrapie [4-6]. Furthermore, previous studies have shown that levels of CDK5 are increased in the brains of AD [7] and HIVE [8] patients, and in scrapie-infected hamsters [6].

In addition to the alterations in synaptic plasticity in mature neurons in these disorders, recent studies have uncovered evidence suggesting that the pathogenic process in humans and animal models of AD and HIV in the brain might include dysregulation of adult neurogenesis [9-14]. This suggests that neurodegeneration may be characterized by not only a loss of mature neurons but also by a decrease in the generation of new neurons in the neurogenic niches of the adult brain. These cell populations that could be targeted include neural progenitor cells (NPCs) in the subventricular zone (SVZ) and in the dentate gyrus (DG) of the hippocampus. Mechanisms of neurogenesis in the fetal brain have been extensively studied, however less is known about the signaling pathways regulating neurogenesis in the adult nervous system and their role in neurodegenerative disorders.

It is clear that the abnormal activation of CDK5 via calpain-mediated cleavage of p35 into the more stable p25 fragment contributes to the pathogenesis of neurodegenerative conditions such as AD and HIVE [4-6,8], however, previous studies have also demonstrated that physiological CDK5 activity is essential for adult neurogenesis [15,16]. Thus, it is possible that abnormal activation of CDK5 and aberrant phosphorylation of its physiological substrates might have detrimental effects on cells residing in the neurogenic niches of the adult brain, and deficits in neurogenesis associated with neurodegeneration might be related to alterations in CDK5 in NPCs. In support of this possibility, we have previously shown that abnormal CDK5 activation impairs neurite outgrowth and neuronal maturation in an *in vitro *model of adult neurogenesis, and in a mouse model of AD-like neurodegeneration and impaired neurogenesis [17]. However, the downstream regulators mediating CDK5-associated defective neurogenesis are unknown.

In this context, CDK5 may mediate alterations in neurogenesis in AD and HIVE via aberrant phosphorylation of its substrates, which include cytoskeletal (neurofilaments, nestin) [18] and synaptic proteins (e.g. synapsin) [19], among others. It is possible that CDK5 substrates implicated in toxicity to mature neuronal populations, such as tau, might be involved, however another possibility is that alternative downstream substrates of CDK5 might modulate neurogenesis and neuronal maturation in the adult hippocampus. Elucidating the signaling pathways and downstream molecular targets involved in the dysregulation of neurogenesis is important to fully understand the mechanisms of neuroplasticity in neurodegenerative disorders.

A subset of physiological substrates of CDK5 includes proteins that have been implicated in neurogenesis (doublecortin, nestin) or neuronal development (collapsin-response mediator protein-2 [CRMP2]). Interestingly, in previous reports, CDK5-mediated phosphorylation of CRMP2 has been linked to neurodegeneration in AD [20-22], and the known role of this protein in growth cone collapse and neuronal development makes it an intriguing candidate for regulating neurogenesis in neurodegenerative disorders. Notably, several recent proteomics-based studies in the brains of human HIV patients have detected differential expression of CRMP2 in human cases with HIVE or HIV-associated dementia [23,24]. Since the role of CRMP2 phosphorylation has not been previously examined in the adult neurogenic niche, or in HIV-associated neurodegenerative disorders (HAND), we sought to better understand the potential function of this protein in the molecular mechanisms involved in CDK5-mediated dysregulation of adult neurogenesis using an *in vitro *model of CDK5 activation in adult hippocampal NPCs [17], and *in vivo *in the brains of patients with HIV and in an animal model of HIV protein neurotoxicity. Here we demonstrate that aberrant phosphorylation of collapsin-response mediator protein-2 (CRMP2) is a critical downstream event contributing to impaired neurite outgrowth in an *in vitro *model of abnormal activation of CDK5 in adult neurogenesis. Using biochemical techniques with genetic and pharmacological manipulation of the p35/CDK5/CRMP2 signaling pathway, we examined the effects of abnormal activation of CDK5 on neurite outgrowth and CRMP2 phosphorylation in NPC-derived neural progeny. We further investigated the contribution of CDK5-mediated hyperphosphorylation of CRMP2 in neurodegenerative processes *in vivo *by evaluating expression and phosphorylation of CRMP2 in the neurogenic niche of human brains with HIVE and in gp120 transgenic (tg) mice. These results reveal a role for CRMP2 in impaired neuronal maturation mediated by abnormal CDK5 activation, and identify a potential downstream functional mechanism involving microtubule destabilization that could contribute to impaired neuronal maturation during adult neurogenesis in neurodegenerative conditions.

## Results

### CRMP2 phosphorylation is increased in an *in vitro *model of adult neurogenesis with abnormal CDK5 activation

**Figure 1:**
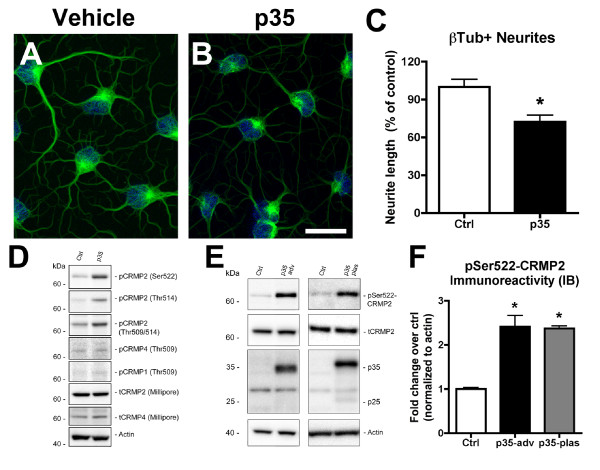
**Impaired neurite outgrowth is accompanied by CRMP2 hyperphosphorylation in NPC-derived neural progeny overexpressing p35**. Differentiating NPCs were grown on glass coverslips and infected on day 2 with an adenoviral vector expressing p35. Cells were processed for immunocytochemistry and immunoblot analyses. For β-tubulin immunofluorescence, on day 4, cells were briefly extracted, then fixed with glutaraldehyde and processed. (A,B) Reduced β-tubulin-immunoreactive neurites in NPC-derived neural progeny infected with p35-adv. (C) Quantitative image analysis showing reduced neurite lengths in NPC-derived neural progeny with p35 expression. (D) Immunoblot analysis of cell lysates from control and p35-expressing NPCs using a panel of antibodies specific for various isoforms and phosphorylated forms of CRMP. (E) Immunoblot analysis of total cell lysates showing increased CRMP2 phosphorylation in NPC-derived neural progeny expressing p35 from adenoviral (left panel) or plasmid (right panel) vectors. (F) Semi-quantitative image analysis of fold change in CRMP2 phosphorylation at the CDK5 epitope (Ser522) in immunoblots from cells expressing p35 from adenoviral or plasmid vectors. * p < 0.05 compared to vehicle-treated controls by one-way ANOVA with post-hoc Dunnett's test. Scale bar = 10 μm.

In order to investigate the molecular mechanisms that might contribute to defective adult neurogenesis in neurodegenerative disorders, we utilized a model system of adult neurogenesis, in which activation of CDK5 is induced by virus-mediated overexpression of the CDK5 activator, p35. We have previously shown that these conditions result in dramatically reduced neurite outgrowth of β-Tubulin-positive processes compared to vehicle-treated controls [17]. At high magnification, cells expressing high levels of p35 had significantly shorter processes and diffuse cytoplasmic β-Tubulin immunoreactivity compared to vehicle-treated controls (Figure 1A-C). This suggests that CDK5 plays a critical role during neuronal maturation of adult hippocampal NPCs, however the downstream mediators of this effect are unknown. It is possible that CDK5 substrates implicated in toxicity to mature neuronal populations, such as tau, might be involved, however it is also possible that alternative targets of CDK5 might modulate neuronal maturation in the adult hippocampus.

CRMP2 has been previously identified as a downstream target of CDK5 and is phosphorylated by this kinase at the Ser522 epitope [25,26]. Although abnormal CDK5 activation has been implicated in neurodegeneration, and CDK5 is critical in the process of adult neurogenesis, the role of CRMP2 in neurogenesis under conditions of abnormal CDK5 activation is unclear. In order to investigate whether CRMP2 might be a target of abnormal CDK5 activity in NPC-derived neural progeny over-expressing p35, we performed western blot screening with a panel of antibodies specific for different phosphorylated forms of CRMP2 and related proteins (CRMP4, CRMP1) or total non-phosphorylated forms (Table 1). This analysis confirmed that CRMP2 was hyperphosphorylated at the CDK5-specific epitope (Ser522) in total cell lysates from NPC-derived neural progeny overexpressing p35 compared to control cultures (Figure 1D-F). Increased phosphorylation was also detected at other residues in the C-terminal tail of CRMP2 (Thr509/Thr514) (Figure 1D), however since previous reports have shown that phosphorylation by CDK5 at the Ser522 residue is required as a priming step before GSK3β-mediated phosphorylation at the nearby Thr residues can occur [25,27,28], we focused our investigations on the CDK5-specific epitope at Ser522. Total levels of CRMP2 were unchanged in cells overexpressing p35 compared to control cells, and other related proteins from the CRMP family were not differentially phosphorylated under these conditions of CDK5 activation (Figure 1D).

**Table 1:** Antibodies tested and reactive epitopes against phosphorylated and total CRMP proteins.

Antibody	Epitope	Company	Species	Reactivity
CRMP2	pThr514	Cell Signaling	Rabbit	Human, mouse, rat

CRMP2	pThr514	Kinasource	Sheep	Human, mouse

CRMP2	pThr509, pThr514	Kinasource	Sheep	Human, mouse, rat

CRMP2	pSer522	ECM Biosciences	Rabbit	Human, mouse, rat

CRMP2	pSer522	Kinasource	Sheep	Human, mouse, rat

CRMP2	pThr555	ECM Biosciences	Rabbit	Human, mouse, rat

CRMP2	total (C-terminus)	ECM Biosciences	Rabbit	Human, mouse, rat

CRMP2	total (peptide)	Millipore	Rabbit	Rat

CRMP2	total (recombinant)	Kinasource	Sheep	Human, mouse CRMP1,2,4

CRMP1	pThr509	Kinasource	Sheep	Human

CRMP4	pThr509	Kinasource	Sheep	Human, mouse, rat

CRMP4 (TUC4)	total (peptide)	Millipore	Rabbit	Human, mouse, rat

For confirmation that the observed increase in CRMP2 phosphorylation was not due to virus-specific effects, we performed similar experiments using an alternative vector for p35 overexpression. For this purpose, we performed immunoblot analysis in parallel with NPC-derived neural cells infected with p35-adv or cells transfected with a plasmid expressing myc-tagged p35. We found that phosphorylation of the CDK5 epitope Ser522-CRMP2 was significantly upregulated in cells overexpressing p35 via both adenoviral vector or plasmid transfection compared to uninfected controls (Figure 1E,F). For all subsequent experiments, results shown are from experiments where untagged p35 was overexpressed using the adenoviral vector.

To further verify that CRMP2 is a target of p35-mediated abnormal activation of CDK5, we used siRNA to knock down expression of the endogenous CRMP2 protein (siCRMP2), and performed immunoblot analysis to assess levels of pSer522-CRMP2 and total CRMP2 (Additional File 1, Figure S1). We found that transfection with siCRMP2 decreased total levels of CRMP2 expression by approximately 80% compared to controls in both untreated and p35-expressing NPC-derived neural progeny (Additional File 1, Figure S1 A,B). Moreover, transfection with siCRMP2 dramatically reduced levels of pSer522-CRMP2 by over 90% in control cells, and completely reversed the hyperphosphorylation of CRMP2 induced by p35 expression in NPC-derived neural progeny (Additional File 1, Figure S1 A,C). Taken together, these studies confirmed that CRMP2 was hyperphosphorylated at the CDK5-specific residue (Ser522) under conditions of abnormal CDK5 activation.

### CRMP2 phosphorylation can be reversed *in vitro *with pharmacological or genetic down-regulation of CDK5

Our previous observations showed that abnormal CDK5 activation in NPC-derived neural progeny impaired neurite outgrowth and cell maturation [17] (Figure 1). Moreover, down-regulating CDK5 activity or expression reversed the neurite outgrowth deficits [17]. In order to determine whether CDK5-mediated CRMP2 phosphorylation might contribute to these effects, we used a pharmacological inhibitor of CDK5 (Roscovitine) or an siRNA-based approach to down-regulate CDK5 activity or expression, respectively, and assessed levels of CRMP2 phosphorylation by immunocytochemistry and immunoblot. For this purpose, differentiating NPCs were pre-treated with Roscovitine, or transfected with siRNA targeting CDK5 for 6 hrs, followed by infection with adenovirus expressing p35. We have previously qualified and tested the siRNA targeting CDK5 for efficacy in our system using two unique siRNA sequences [17].

**Figure 2:**
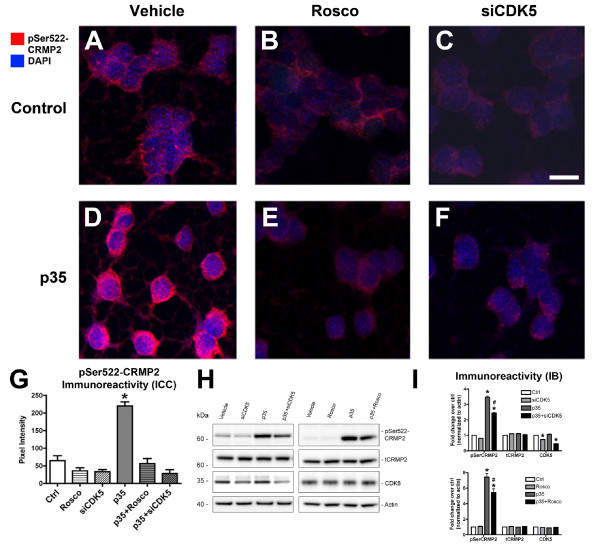
**Down-regulation of CDK5 reduces CRMP2 phosphorylation in NPC-derived neural progeny**. Differentiating NPCs were treated on day 2 with the pharmacological inhibitor Roscovitine (Rosco) or siRNA against CDK5 (siCDK5), followed by infection with p35-adenovirus. On day 4, NPC-derived neural progeny were fixed for immunofluorescence or lysed for immunoblot analysis. (A-C) Immunocytochemical analysis with an antibody against pSer522-CRMP2 showed low levels of endogenous immunoreactivity in uninfected, vehicle-treated cells (*A*), with a slight reduction in cells treated with Roscovitine (*B*) or siCDK5 (*C*). (D-F) NPC-derived neural progeny expressing p35 showed a notable increase in CRMP2 phosphorylation (*D*) that was reversed by treatment with Rosco (*E*) or siCDK5 (*F*). (G) Reversal of CRMP2 hyperphosphorylation with CDK5 down-regulation. (H) Immunoblot analysis of pSer522-CRMP2, total (t)CRMP2, CDK5, and actin as a loading control. (I) Semi-quantitative image analysis of levels of CRMP2 phosphorylation in NPCs infected with p35-adv with or without Rosco or siCDK5. * p < 0.05 compared to vehicle-treated controls by one-way ANOVA with post-hoc Dunnett's test. # p < 0.05 compared to p35-expressing NPCs by one-way ANOVA with post-hoc Tukey-Kramer test. Scale bar = 10 μm.

Notably, under conditions of reduced CDK5 activity or expression in p35-expressing NPC-derived neural progeny, reduced levels of CRMP2 phosphorylation were detected, as demonstrated by immunocytochemical (Figure 2A-G) and immunoblot analyses (Figure 2H,I) with an antibody against pSer522-CRMP2. Roscovitine treatment in the absence of p35 over-expression does not appear to have a significant effect on levels of CRMP2 phosphorylation (Figure 2). It is possible that this could be due to alternative kinases phosphorylating CRMP2, however as CDK5 is the only kinase known at this time to phosphorylate CRMP2 at Ser522, another mechanism must be at play. Previous studies have shown that Ser522-phosphorylated CRMP2 is highly resistant to desphosphorylation by protein phosphatases [29]. Therefore, it is likely that the CRMP2 that is already phosphorylated by basal CDK5 activity prior to roscovitine treatment remains phosphorylated and stable, while roscovitine treatment of cells with exogenous p35 expression more effectively modulates levels of CRMP2 phosphorylation at the Ser522 residue. Taken together, these results support the possibility that abnormal CDK5 activation targets CRMP2 for hyperphosphorylation, and this can be reversed by down-modulating abnormal CDK5 activity.

### Expression of a non-phosphorylatable CRMP2 construct reduces CRMP2 phosphorylation and rescues neurite outgrowth defects in adult NPCs with abnormal CDK5 activation

In order to determine whether CRMP2 phosphorylation is directly related to the neurite outgrowth defects observed in our cellular model of abnormal CDK5 activation in adult neurogenesis, we utilized site-directed mutagenesis to generate a construct encoding a mutant (S522A) form of CRMP2 (S522A-CRMP2) that is not phosphorylatable by CDK5 at the Ser522 epitope (Additional File 1, Figure S2 A). Immunoblot analysis confirmed that transfection of differentiating NPCs with the S522A-CRMP2 construct or WT-CRMP2 resulted in similar levels of total CRMP2 expression (Additional File 1, Figure S2 B). In contrast, levels of pSer522-CRMP2 immunoreactivity were considerably lower in NPC-derived neural progeny expressing S522A-CRMP2 compared to WT-CRMP2-transfected controls (Additional File 1, Figure S2 B,C). Controls including transfection agent (Lipofectamine) alone or CMV-GFP vector control showed that levels of pSerCRMP2 and total CRMP2 immunoreactivity were similar to non-transfected controls (Additional File 1, Figure S2 B,C).

**Figure 3:**
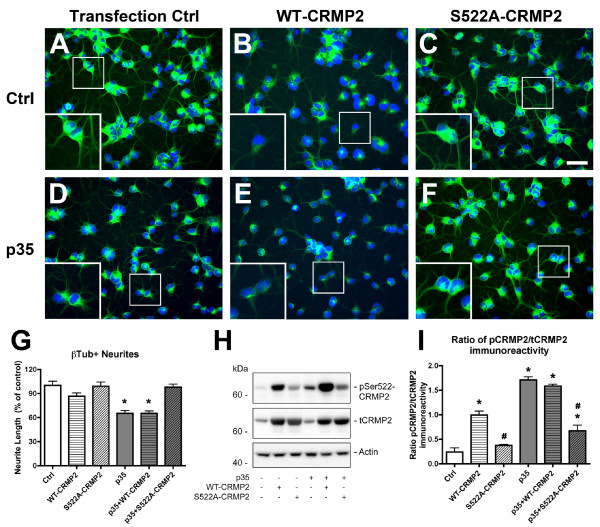
**Expression of S522A-CRMP2 mutant construct rescues neurite deficits in p35-expressing NPC-derived neural progeny**. Differentiating NPCs were transfected on day 2 with wild-type (WT) human CRMP2 or mutated S522A-CRMP2 plasmids, followed by infection with p35 adenovirus. Cells were fixed on day 4 of differentiation with glutaraldehyde for β-tubulin immunofluorescence, or lysed for immunoblot analysis. (A-F) Immunocytochemical analysis of β-tubulin immunoreactivity showed reduced β-tubulin-positive neurites in NPC-derived neural progeny infected with p35-adv with or without co-expression of WT-CRMP2, and rescue with co-expression of S522A-CRMP2 plasmid. (G) Image analysis showing reduced β-tubulin-positive neurite lengths in p35-expressing and p35+WT-CRMP2 NPC-derived neural progeny that was recovered after co-expression of p35 and S522A-CRMP2. (H) Immunoblot analysis showing levels of CRMP2 phosphorylation in p35-expressing NPC-derived neural progeny transfected with S522A-CRMP2 or WT-CRMP2 plasmid. (I) Semi-quantitative image analysis of ratios of pSer522-CRMP2 (pCRMP2)/total CRMP2 (tCRMP2) levels in NPC-derived neural progeny expressing p35 with and without WT-CRMP2 or S522A-CRMP2 co-expression. * p < 0.05 compared to vehicle-treated controls by one-way ANOVA with post-hoc Dunnett's test. # p < 0.05 compared to WT-CRMP2-transfected NPCs by one-way ANOVA with post-hoc Tukey-Kramer test. Scale bar = 25 μm, 10 μm (insets).

In order to assess whether down-regulating CDK5-mediated phosphorylation of CRMP2 at Ser522 by expression of a mutant construct might have a protective effect on the neurite alterations observed in NPC-derived neural progeny with abnormal CDK5 activity, neurite outgrowth analysis was performed in cultured cells immunolabeled with an antibody against β-Tubulin (Figure 3A-F). This demonstrated that expression of the mutant S522A-CRMP2 construct rescued the neurite deficits in NPC-derived neural progeny expressing p35, whereas expression of the WT-CRMP2 construct was not protective under these conditions (Figure 3A-G).

Immunoblot analysis confirmed that expression of S522A-CRMP2 reduced levels of CRMP2 phosphorylation in p35-expressing NPC-derived neural progeny compared to WT-CRMP2. We detected a slight apparent increase in pSer-CRMP2 immunoreactivity in lysates from cells expressing the non-phosphorylatable mutant S522A-CRMP2 construct. Higher resolution gel electrophoresis studies indicated that while CRMP2 hyperphophorylation induced by p35 overexpression resulted in a gel mobility shift that was consistent with a change in phosphorylation state [27], no such shift was detected with overexpression of the S522A-CRMP2 mutant construct (data not shown). It is possible that the apparent increase in immunoreactivity with S522A-CRMP2 over-expression could be due to a mild change in protein folding induced by the mutation, causing a slight affinity of the pSerCRMP2 antibody for the mutant unphosphorylated human protein. Levels of total CRMP2 were similar in cells transfected with either the WT or mutant constructs (Figure 3H,I). Levels of CRMP2 phosphorylation were expressed as ratios of pCRMP2/tCRMP2 immunoreactivity because overexpression of the CRMP2 constructs increases total levels of CRMP2. Since over-expression of WT-CRMP2 is known to promote neurite outgrowth in neuronal cell lines [30], when WT-CRMP2 is overexpressed and phosphorylation is increased, there are increased quantities of both total and phospho-CRMP2. Thus, under conditions of CRMP2 overexpression, the ratio of phospho-CRMP2/total-CRMP2 may be the most accurate representation of relative CRMP2 phosphorylation that impacts neurite outgrowth. Taken together, these data support the possibility that phosphorylation of CRMP2 by CDK5 at the Ser522 epitope contributes to impaired neurite outgrowth under conditions of abnormal CDK5 activation in cultured NPCs.

### Tubulin distribution and microtubule organization are disturbed in NPC-derived neural progeny with abnormal CDK5 activation

**Figure 4:**
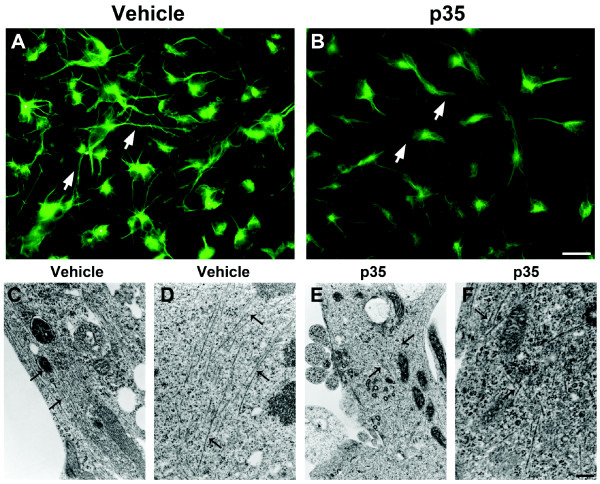
**Live-cell imaging of polymerized tubulin distribution, and ultrastructural analysis of microtubules in NPC-derived neural progeny with CDK5 activation**. Differentiating NPCs were infected on day 2 with an adenoviral vector expressing p35. For live imaging of tubulin distribution, on day 4, cells on coverslips were incubated with Tubulin Tracker Green to stain polymerized tubulin. Coverslips were imaged using a fluorescent microscope. For electron microscopy analysis, NPCs were grown on coverslips embedded in 35 mm dishes, fixed in glutaraldehyde and processed for electron microscopic analysis. (A,B) Live-cell imaging of polymerized tubulin stained with Tubulin Tracker in cell bodies and processes of NPC-derived neural progeny infected with p35-adv. (A) Under control conditions, polymerized tubulin is apparent in bundled microtubule structures in cell processes (arrows). (B) Under conditions of abnormal CDK5 activation, polymerized tubulin staining was primarily detected in diffuse, cytoplasmic fibers (arrows). (C,D) Electron microscopy images showing organized cytoskeletal structure in control NPC-derived neural progeny with long, parallel tubule structures (arrows) along the processes of cells. (E,F) NPC-derived neural progeny expressing high levels of p35 displayed a disorganized cytoskeleton with less abundant, short and web-like filamentous structures (arrows) in the processes of cells. Scale bar = 30 μm (A,B); 0.5 μm (C,E); 0.25 μm (D,F).

Previous studies have shown that phosphorylation of CRMP2 regulates its interactions with tubulin heterodimers [30]. In light of our observations thus far, it is possible that CDK5-mediated hyperphosphorylation of CRMP2 in our *in vitro *model might impair neurite outgrowth by disrupting microtubule polymerization in developing neuronal processes. First, to assess the distribution of polymerized tubulin in live cell cultures, Tubulin Tracker reagent was applied to NPC-derived neural progeny after four days of differentiation under control and activated CDK5 conditions (Figure 4A,B). In cultures infected with p35-adv, reduced neurite complexity was apparent compared to uninfected controls. Furthermore, under control conditions, the distribution of polymerized tubulin appeared to be localized to cell processes, and characterized by well-defined bundled tubulin fibers (Figure 4A). In contrast, under conditions of abnormal CDK5 activation, the polymerized tubulin pool appeared to be comprised of more diffuse, fine, mesh-like tubulin fibers localized to the cytoplasm in cells infected with p35-adv (Figure 4B).

To further investigate the underlying ultrastructural basis of the neurite alterations observed in p35-overexpressing NPC-derived neural progeny, cells on coverslips were fixed in glutaraldehyde and analyzed by electron microscopy (Figure 4C-F). These studies showed that control NPC-derived neural progeny displayed long electron-dense microtubule structures distributed along neuritic processes in a parallel, organized arrangement (Figure 4C,D). In contrast, in the processes of p35-expressing NPC-derived neural progeny, filamentous and tubule-like structures were less abundant, and those that were observed were shorter and more disorganized than in control cells (Figure 4E,F). These results support the possibility that abnormal activation of CDK5 and hyperphosphorylation of CRMP2 during neurogenesis in neurodegenerative disorders might impair microtubule polymerization and disrupt the organization of these important structural components of the cytoskeleton.

### NPC-derived neural progeny with abnormal CDK5 activation show impaired microtubule polymerization following exposure to microtubule destabilizing agents

**Figure 5:**
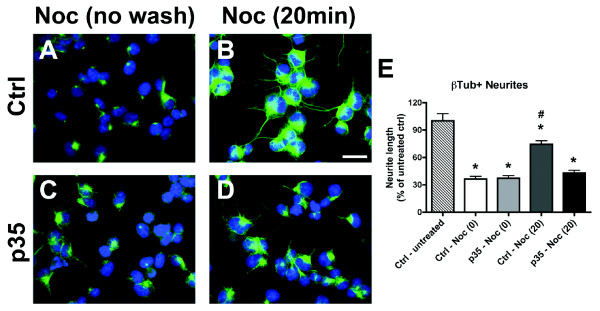
**Tubulin polymerization is impaired in p35-expressing NPC-derived neural progeny following chemical microtubule disruption**. For chemical disruption of microtubules, cultures were treated with 5 μg/mL nocodazole (Noc) for 3 hr, followed by washout and incubation in fresh differentiation media for 20 mins. NPC-derived neural progeny were fixed with glutaraldehyde and processed for β-tubulin immunofluorescence. (A,B) Chemically-induced disruption of microtubule structure in nocodazole-treated control NPC-derived neural progeny (*A*) was recovered by 20 mins post-washout (*B*). (C,D) Disruption of microtubule structure in nocodazole-treated p35-expressing NPC-derived neural progeny (*C*) appeared unchanged by 20 mins post-washout (*D*). (E) Quantitative image analysis of neurite outgrowth in NPC-derived neural progeny following treatment with nocodazole. Untreated control is shown for comparison to baseline neurite lengths. * p < 0.01 compared to vehicle-treated controls by one-way ANOVA with post-hoc Dunnett's test; # p < 0.01 compared to nocodazole-treated controls by one-way ANOVA with post-hoc Tukey-Kramer test. Scale bar = 15 μm.

To further investigate the process of microtubule polymerization in NPC-derived neural progeny, we performed a series of nocodazole washout experiments to visualize the formation of microtubules over a period of 10-30 mins. For this purpose, NPC-derived neural progeny were incubated with nocodazole for 3 hrs, followed by no washout, or washout for 10-30 mins (Figure 5A-D). Cells were permeabilized during glutaraldehyde fixation to allow visualization of intact microtubules, then processed for β-tubulin immunofluorescence and imaged on a fluorescent microscope. This showed, as expected, that directly following nocodazole treatment (no washout), both control and p35-expressing NPC-derived neural progeny displayed a dramatic reduction of β-tubulin immunoreactivity, and microtubule structure was almost completely ablated in both groups (Figure 5A,C). Twenty minutes after nocodazole washout, in both control and NPC-derived neural progeny expressing high levels of p35, we observed the appearance of perinuclear microtubule organizing centers (Figure 5B,D).

By 20 minutes post-washout, control nocodazole-treated cultures (Figure 5B) regained approximately 75% of the neurite lengths compared to untreated control cultures (Figure 5E), while p35-expressing cells only displayed diffuse β-tubulin immunoreactivity in processes (Figure 5D), and no significant increase in neurite lengths (Figure 5E). Only very fine processes were detected under these conditions up to 30 minutes post-washout (data not shown). These results are consistent with the possibility that abnormal activation of CDK5 in the pathogenesis of neurodegenerative disorders might disrupt adult neurogenesis by impairing microtubule polymerization and neurite outgrowth.

### Increased CRMP2 phosphorylation in a model of HIV-protein mediated activation of CDK5 in adult neurogenesis, and reversal with CDK5 siRNA

**Figure 6:**
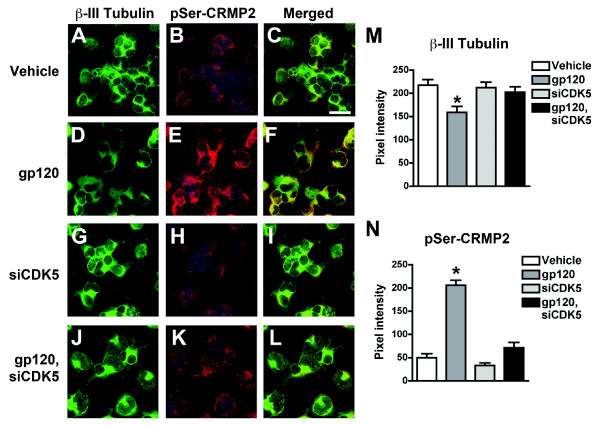
**Increased CRMP2 phosphorylation in NPCs treated with HIV-gp120 protein, and rescue with CDK5 siRNA knockdown**. Differentiating NPCs were transfected day 2 of differentiation with siRNA specific for CDK5 (siCDK5) or transfection reagent control, followed by treatment with recombinant HIV-gp120 protein (100 ng/mL) or vehicle control. Cells were fixed on day 4 of differentiation for double-immunolabeling analysis with antibodies against β-III Tubulin (immature neuronal marker) and pSer522-CRMP2 (pSer-CRMP2). (A-C) β-III Tubulin-positive NPC-derived neuronal progeny treated with vehicle control express background levels of pSer-CRMP2. (D-F) NPC-derived neuronal progeny treated with gp120 for 48 hrs display increased pSer-CRMP2 immunoreactivity in β-III Tubulin-positive cells. (G-I) β-III Tubulin-positive NPC-derived neuronal progeny treated with siCDK5 show low levels of pSer-CRMP2 immunoreactivity. (J-L) β-III Tubulin-positive NPC-derived neuronal progeny treated with siCDK5 and gp120 show background levels of pSer-CRMP2 immunoreactivity. (M, N) Semi-quantitative image analysis of β-III Tubulin (M) and pSer-CRMP2 (N) immunoreactivity. * p < 0.05 compared to vehicle-treated controls by one-way ANOVA with post-hoc Dunnett's test. Scale bar = 20 μm.

We and others have shown that CDK5 signaling is dysregulated in patients with HIVE and in animal and *in vitro *models of HIV-associated neurotoxicity [8,31]. These studies demonstrate that the HIV protein gp120 contributes to calpain-mediated activation of CDK5 and downstream hyperphosphorylation of CDK5 substrates such as tau in mature neuronal populations [8,31]. In this context, we sought to examine the possibility that HIV proteins could modulate CRMP2 phosphorylation in a similar manner to what we observed in our *in vitro *model of p35-directed abnormal CDK5 activation in adult neurogenesis. For this purpose, NPC-derived neural progeny were exposed to recombinant HIV-gp120 protein for 24 hrs on day 3 of differentiation. Double-immunolabeling analysis with antibodies against the immature neuronal marker β-III Tubulin and pSer522-CRMP2 revealed that gp120 treatment resulted in upregulated CRMP2 phosphorylation, and this effect was accompanied by reduced β-III Tubulin immunoreactivity (Figure 6A-F,M,N). siRNA experiments to knock down CDK5 expression showed that these effects could be rescued by down-modulating CDK5 expression (Figure 6G-N). Similar to what we observed previously using the CDK5-targeting siRNA (Figure 2 and [17]), in NPCs expressing CDK5 siRNA alone or in combination with gp120 treatment, CDK5 levels were reduced by approximately 50-60% (data not shown). Taken together, these studies in an *in vitro *model of adult neurogenesis reveal that abnormal activation of CDK5 mediated by either p35 overexpression or HIV-gp120 treatment triggers hyperphosphorylation of CRMP2, which may contribute to microtubule destabilization and impaired neurogenesis and neuronal maturation in the mature brain.

### CRMP2 phosphorylation is increased and dendritic maturation is impaired in primary neuronal cultures with abnormal CDK5 activation

**Figure 7:**
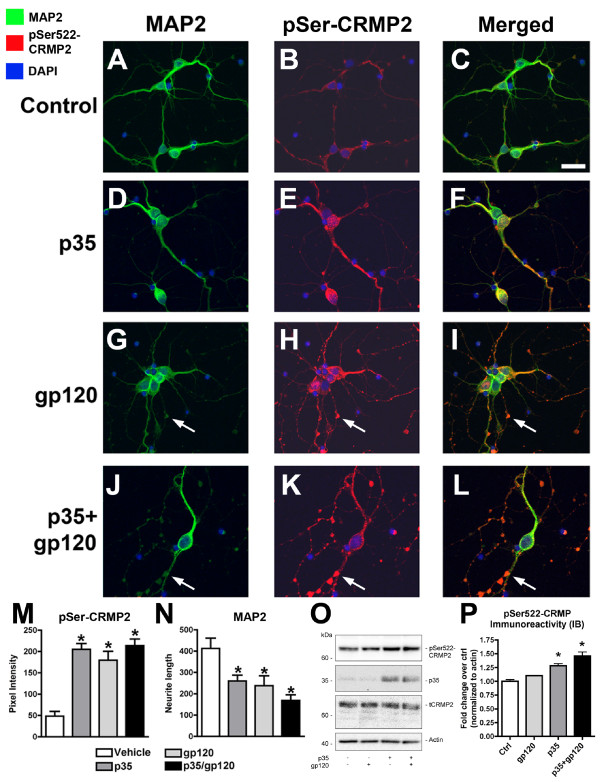
**CRMP2 is hyperphosphorylated and dendritic development is impaired in primary neurons over-expressing p35 alone or in combination with HIV-gp120 protein treatment**. Primary rat hippocampal neurons were infected two days after plating with p35-adv, followed by treatment with recombinant HIV-gp120 protein or vehicle control. Cells were fixed on day 4 of differentiation for double-immunolabeling analysis with antibodies against MAP2 (dendritic marker) and pSer522-CRMP2 (pSer-CRMP2). (A-C) MAP2-positive primary neurons treated with vehicle control express background levels of pSer-CRMP2. (D-F) Primary neurons infected with p35-adv display increased pSer-CRMP2 immunoreactivity and reduced MAP2-immunoreactive dendrites. (G-I) Primary neurons treated with recombinant HIV-gp120 display increased pSer-CRMP2 immunoreactivity and reduced MAP2-immunoreactive dendrites, with an accumulation of pSer-CRMP2 immunoreactivity in varicosities along the neuronal processes (arrows). (J-L) Primary neurons infected with p35-adv and treated with recombinant HIV-gp120 display increased pSer-CRMP2 immunoreactivity and extensive damage to MAP2-immunoreactive dendrites, with an abundant accumulation of pSer-CRMP2 immunoreactivity in varicosities along the neuronal processes (arrows). (M, N) Semi-quantitative image analysis of pSer-CRMP2 (M) and MAP2 (N) immunoreactivity. (O) Immunoblot analysis of total cell lysates showing increased CRMP2 phosphorylation in primary hippocampal neurons infected with p35-adv or treated with HIV-gp120 protein. (P) Semi-quantitative image analysis of fold change in CRMP2 phosphorylation at the CDK5 epitope (Ser522) in immunoblots from cells expressing p35-adv or treated with gp120. * p < 0.05 compared to vehicle-treated controls by one-way ANOVA with post-hoc Dunnett's test. Scale bar = 20 μm.

Since our results in NPC-derived neural progeny suggest that abnormal CDK5 activation and hyperphosphorylation of CRMP2 contributes to impaired neurite outgrowth during neurogenesis, we sought to determine whether these factors might also play a role in neurite maturation in mature neuronal cells. For this purpose, we infected primary rat hippocampal neurons with p35-adv, and two days later, levels of CRMP2 phosphorylation and dendritic integrity were analyzed by immunocytochemistry and immunoblot. Similar to NPC-derived neural progeny, primary hippocampal neurons overexpressing p35 displayed increased pSer522-CRMP2 immunoreactivity compared to uninfected controls (Figure 7A-F, M-P). Interestingly, under control conditions, primary neurons expressed higher levels of pSer522-CRMP2 than NPCs, and thus the change in pSer522-CRMP2 immunoreactivity with p35 overexpression was less dramatic in primary neuronal cultures than in NPCs (40-50% increase in CRMP2 phosphorylation in primary neurons compared to 3-4 fold in NPC-derived neural progeny). However, similar to what was observed in NPCs, in primary neurons increased p35 expression and CRMP2 phosphorylation was accompanied by reduced neurite maturation and dendritic complexity, as shown by immunolabeling with an antibody against microtubule-associated protein-2 (MAP2, dendritic marker) (Figure 7A-F, M, N). Similar effects were observed in primary neuronal cultures exposed to HIV-gp120 protein for 24 hrs alone or in combination with p35 overexpression (Figure 7G-P). Under these conditions, extensive damage to MAP2-immunoreactive processes was visible, and pSer522-CRMP2 immunoreactive deposits were detected in varicosities along the neuronal processes (Figure 7G-L). Taken with the observations from experiments with NPC-derived neural progeny, these results suggest that abnormal CDK5 activation and subsequent CRMP2 hyperphosphorylation may have deleterious effects on neurite outgrowth in both immature and mature neuronal populations in the adult brain. Thus, the role of CRMP2 hyperphosphorylation in neurodegenerative disorders where abnormal CDK5 activation contributes to the pathogenesis of the disease warrants investigation.

### CRMP2 is hyperphosphorylated in the brains of patients with HIVE

**Figure 8:**
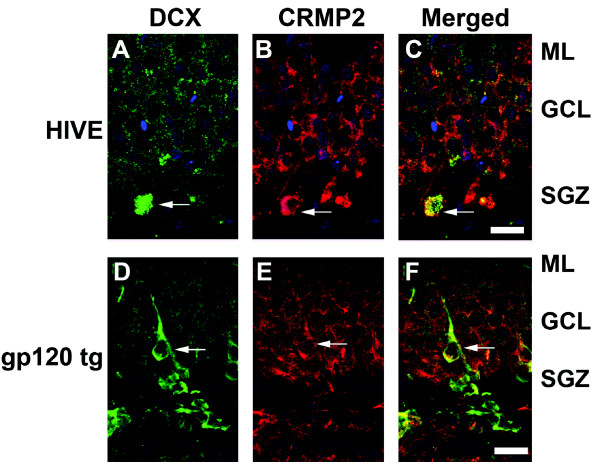
**Patterns of CRMP2 immunoreactivity in the hippocampus of an HIV encephalitis brain and a gp120 transgenic mouse brain**. Forty μm-thick vibratome sections from the brains of HIV encephalitis (HIVE) patients and gp120 transgenic (tg) mice were processed for double-immunolabeling analysis with antibodies against doublecortin (DCX) and CRMP2. (A-C) Intracellular CRMP2 immunoreactivity was detected throughout cells in the granular cell layer (GCL) and into the molecular layer (ML) of the hippocampal dentate gyrus in a representative section from the brain of a patient with HIVE. A subset of DCX-positive cells in the subgranular zone (SGZ) co-expressed CRMP2 (arrows). (D-F) Intracellular CRMP2 immunoreactivity was detected throughout cells in the granular cell layer (GCL) and into the molecular layer (ML) of the hippocampal dentate gyrus in a representative section from the brain of a gp120 tg mouse. A subset of DCX-positive cells in the subgranular zone (SGZ) co-expressed CRMP2 (arrows). Scale bar = 15 μm.

To further evaluate the potential contribution of CRMP2 to neurogenic alterations in the pathogenesis of HIV in the brain, we examined expression levels and patterns of immunoreactivity of CRMP2 in the hippocampus of HIV patients with and without encephalitis (patient demographics and clinic-pathological characteristics presented in Table 2). In support of a role for CRMP2 in the neurogenic niche of the adult hippocampus, double-labeling immunocytochemical analysis with antibodies against doublecortin (DCX, a marker of immature neuroblasts) and CRMP2 revealed that CRMP2 is expressed throughout the hippocampal DG in the brains of patients with HIVE (Figure 8A-C). A subset of cells in the subgranular zone (SGZ) co-expressed both DCX and CRMP2, suggesting that endogenous neuronal progenitor cells in the adult hippocampus could be sensitive to CDK5-mediated hyperphosphorylation of CRMP2 (Figure 8A-C).

**Table 2:** Demographic and clinico-pathological characteristics of HIV+ cases with and without HIV encephalitis.

Neuropathology diagnosis	Age	Gender	PM time (hrs)	Brain weight	Cause of Death
Normal	55	M	25	1220	Heart failure

Normal	53	M	18	1410	Respiratory failure

Normal	47	M	12	1200	Bronchopneumonia

Normal	35	M	9	1440	Hypotensive shock

Normal	36	F	24	1370	Respiratory failure

Normal	49	M	30	1350	Cryptococcal pneumonia

Normal	54	M	14	1220	Cardiac infarction

Normal	67	M	15	1440	Complications of HIV and HCV co-infection

HIV encephalitis	34	M	30	1120	Heart failure

HIV encephalitis	39	M	19	1370	Organ failure

HIV encephalitis	37	M	5	1510	Respiratory failure

HIV encephalitis	55	M	15	1040	Bacterial pneumonia

HIV encephalitis	41	M	12	1230	Cachexia

HIV encephalitis	43	M	6	1350	AIDS

HIV encephalitis	47	M	47	1260	Sepsis

HIV encephalitis	43	M	72	1410	Undetermined

PM = post-mortem time, HCV = Hepatitis C virus

**Figure 9:**
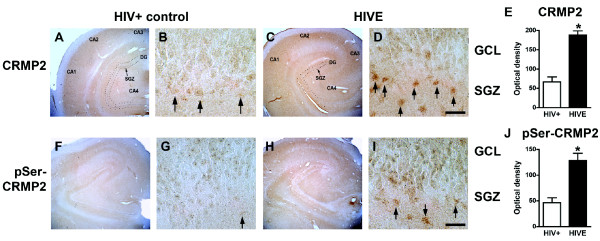
**Increased CRMP2 expression and phosphorylation in the hippocampus of patients with HIV encephalitis**. Forty μm-thick vibratome sections from the hippocampus of HIV+ control and HIV encephalitis (HIVE) patients were processed for immunolabeling analysis with antibodies against CRMP2 and pSer522-CRMP2 (pSer-CRMP2). Low power images show overall immunoreactivity throughout the hippocampus, including the CA1-4 regions, the dentate gyrus (DG) and the subgranular zone (SGZ), and high power images show more detail of the DG including the SGZ and granular cell layer (GCL). (A-D) Patterns of total CRMP2 immunoreactivity in the hippocampus from representative HIV+ non-encephalitis (control) and HIVE brains at low (A, C) and high (B, D) power. Numerous intensely immunoreactive cell profiles were detected in the SGZ (arrows), particularly in the brains of HIVE patients. (E) Semi-quantitative image analysis of CRMP2 immunoreactivity in the hippocampal DG of HIV patients. (F-I) Patterns of pSer-CRMP2 immunoreactivity in the hippocampus from representative HIV+ non-encephalitis (control) and HIVE brains at low (F, H) and high (G, I) power. (J) Semi-quantitative image analysis of pSer-CRMP2 immunoreactivity in the hippocampal DG of HIV patients. * p < 0.05 compared to HIV+ controls by unpaired two-tailed Student's t-test (n = 8 per group). Scale bar = 200 μm (A,C,F,H); 30 μm (B,D,G,I).

To determine whether CRMP2 phosphorylation or expression is altered in the brains of patients with HIVE, immunocytochemical analysis was performed with antibodies against total CRMP2 and pSer522-CRMP2 in hippocampal sections from HIV+ (no encephalitis) and HIVE brains (Figure 9). Both total CRMP2 and pSer522-CRMP2 displayed a granular expression pattern in the granular cell layer and subgranular zone of the hippocampal dentate gyrus. Consistent with our previous report that CDK5 expression and activity is increased in the brains of patients with HIVE [8], image analysis revealed that overall CRMP2 and phosphorylated CRMP2 (Ser522 epitope) levels were significantly increased in the hippocampus of HIVE brains compared to HIV+ controls without encephalitis (Figure 9A-J). Numerous intensely CRMP2-immunoreactive nuclear profiles were also detected in the SGZ of HIVE brains (Figure 9D,I), suggesting that in the pathogenesis of HIVE, cells in this neurogenic region are sensitive to CDK5-mediated upregulation of CRMP2 phosphorylation.

To further examine overall expression levels of CRMP2 in the brains of HIVE patients, immunoblot analysis was performed with brain homogenates from HIV+ (no encephalitis) controls and HIVE patients (Additional File 1, Figure S3). This analysis revealed that levels of phosphorylated (Ser522) CRMP2 were significantly upregulated in HIVE patients compared to non-encephalitic HIV+ controls (Additional File 1, Figure S3 A,B). In fact, while some non-encephalitis cases displayed only very low levels of immunoreactivity with the pSer522-CRMP2 antibody, every one of the HIVE cases examined showed robust pSer522-CRMP2 immunoreactivity by immunoblot (Additional File 1, Figure S3 A).

### Hyperphosphorylated CRMP2 is detected in the brains of gp120 tg mice, and is reversed with genetic down-modulation of CDK5 expression

**Figure 10:**
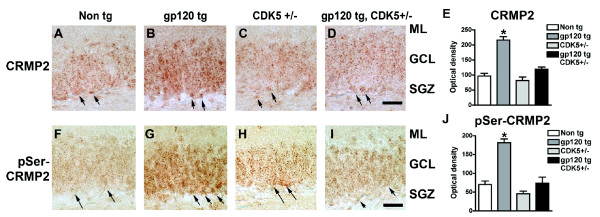
**Increased CRMP2 phosphorylation in gp120 transgenic mice is rescued with down-modulation of CDK5 expression**. Forty μm-thick vibratome sections from the brains of 8-month old non-transgenic (nontg), gp120 transgenic (tg), CDK5 heterozygous-deficient (CDK5+/-), and gp120 tg/CDK5+/- mice were processed for immunolabeling analysis with antibodies against CRMP2 and pSer522-CRMP2 (pSer-CRMP2). (A-D) Patterns of total CRMP2 immunoreactivity in the dentate gyrus from representative mouse brains showing a punctate expression pattern throughout the granular cell layer (GCL) and subgranular zone (SGZ), with background immunoreactivity detected in the molecular layer (ML). Intensely immunoreactive cell profiles were detected in the SGZ (arrows), particularly in the brains of gp120 tg mice. (E) Semi-quantitative image analysis of CRMP2 immunoreactivity in the hippocampal DG of nontg, gp120 tg, CDK5+/- and gp120 tg/CDK5+/- mice. (F-I) Patterns of pSer-CRMP2 immunoreactivity in the hippocampal DG from representative mouse brains showing a punctate expression pattern throughout the granular cell layer (GCL) and subgranular zone (SGZ), with background immunoreactivity detected in the molecular layer (ML). Intensely immunoreactive cell profiles were detected in the SGZ (arrows), particularly in the brains of gp120 tg mice. (J) Semi-quantitative image analysis of pSer-CRMP2 immunoreactivity in the hippocampus of nontg, gp120 tg, CDK5+/- and gp120 tg/CDK5+/- mice. * p < 0.05 compared to nontg controls by one-way ANOVA with post-hoc Dunnett's test (n = 4 per group). Scale bar = 30 μm.

Similar to what we observed in the hippocampus of HIVE patients, CRMP2 and pSer522-CRMP2 immunoreactivity were detected throughout the DG in immunolabeled sections from the brains of non-transgenic (nontg) and gp120 tg mice (Figure 10). In support of a role for CRMP2 in this neurogenic region, double-labeling immunocytochemical analysis with antibodies against DCX and CRMP2 revealed that CRMP2 is expressed throughout the hippocampal DG in the brains of gp120 tg mice (Figure 8D-F). Levels of total CRMP2 and pSer522-CRMP2 immunoreactivity were increased in the hippocampus of gp120 tg mice compared to nontg controls (Figure 10A,B,E,F,G,J), indicating that CDK5 activation in this model contributes to abnormal phosphorylation of CRMP2.

In our *in vitro *model of abnormal CDK5 activation in NPC-derived neural progeny, we found that CDK5-directed CRMP2 hyperphosphorylation could be reversed by knocking down CDK5 expression or activity (Figure 2). Therefore, we sought to determine if this effect could also be rescued *in vivo *in gp120 tg mice crossed with a CDK5 heterozygous-deficient (CDK5+/-) line. Immunocytochemical analysis revealed that this was the case, as gp120 tg/CDK5+/- animals displayed levels of CRMP2 (Figure 10A-E) and pSer522-CRMP2 (Figure 10F-J) immunoreactivity that were comparable to nontg controls.

## Discussion

Abnormal CDK5 activity has been shown to contribute to neurotoxicity during the pathogenesis of several neurodegenerative disorders, including HIV neurotoxicity, AD, and prion-related encephalopathies. In the progression of these disorders, activation of CDK5 may trigger a cascade of hyperphosphorylation of downstream targets, and subsequent modulation of critical cellular functions. Most studies have focused on the role of tau hyperphosphorylation in mediating the neurodegenerative effects of CDK5 and other aberrantly activated kinases in mature neurons [8,32,33]. However, considering the myriad substrates phosphorylated by CDK5, other downstream targets may be involved, particularly in distinct functions such as neurogenesis and neuronal maturation. In this context, we report that dysregulation of the growth cone signaling protein CRMP2 by abnormal CDK5 activation in an *in vitro *model of adult neurogenesis contributes to defective neurite outgrowth during neuronal maturation. Down-regulation of CDK5 activity with the pharmacological inhibitor Roscovitine or siRNA knockdown, or expression of a non-phosphorylatable CRMP2 mutant construct (S522A) rescued the neurite defects associated with abnormal activation of CDK5 in NPC-derived neural progeny.

In support of a role for CRMP2 in neurodegenerative pathways, previous studies have revealed that CRMP2 is hyperphosphorylated as a result of abnormal CDK5 activity in the brains of AD patients and in a transgenic mouse model of AD [20]. In AD brains, phosphorylated CRMP2 associates with damaged neurites and neurofibrillary tangles [21,22,34], and accumulates in neurons surrounding cortical amyloid plaques [35]. More recently, several proteomics-based studies in the brains of human HIV patients have identified differential expression of CRMP2 in cases with HIVE or HIV-associated dementia [23,24]. The present study reveals that levels of total and phosphorylated (Ser522) CRMP2 were significantly increased in neurogenic sites of the hippocampus from HIVE patients compared to non-encephalitic HIV+ controls, and similar results were observed in a mouse model of HIV-gp120 neurotoxicity. While the present study was focused on the effects of CDK5 activation and CRMP2 alterations in the neurogenic region of the hippocampus, it is of interest that we observed cells throughout the SGZ and the granular cell layer displaying increased CRMP2 immunoreactivity and phosphorylation, particularly in the gp120 tg mouse model. This suggests that mature neuronal cell populations in addition to NPCs may be vulnerable to CDK5-mediated abnormal CRMP2 phosphorylation. Our results in primary hippocampal neurons also support this possibility, however future studies in other mature neuronal cell types and alternative brain regions (e.g. cortex) will be necessary to elucidate whether CDK5-mediated alterations in CRMP2 is a common pathological feature of HIV-associated neurodegeneration.

In the tg mouse model, hippocampal CRMP2 hyperphosphorylation was reversed with genetic down-modulation of CDK5. Since CDK5 activity and phosphorylation of other substrates in mature neurons is increased in models of HIV neurotoxicity [8], under these conditions, heterozygous CDK5 deficiency may be protective by normalizing the activity of CDK5 via moderate reduction of CDK5 expression levels. In support of this possibility, we have previously demonstrated that heterozygous CDK5 deficiency reverses hyperphosphorylation of tau in mature neurons in the frontal cortex of gp120 tg mice [8], and that CDK5 knock-down rescues neurogenic deficits in an animal model of AD [17]. The present study suggests that hyperphosphorylation of CRMP2 may be an important feature of HIV-associated neurodegeneration, and identifies a potential link between abnormal activation of CDK5 and impaired neurogenesis that has been observed in animal models of HIV neurotoxicity [36], however future studies will be necessary to elucidate whether CRMP2 hyperphosphorylation plays a direct causative role in neurogenic or neurodegenerative alterations in the pathogenesis of HAND.

Given that CRMP2 is a known substrate of CDK5, along with the recent evidence that CDK5 and CRMP2 are dysregulated in HIVE [8,23,24], activation of CDK5 and subsequent hyperphosphorylation of CRMP2 may be an important mediator of neurodegeneration in HIV infection. The results of the present study also reveal a new potential role for CRMP2 alterations in the mechanisms of defective adult neurogenesis in neurodegenerative disorders such as HIVE. This is in line with previous studies that have shown defective neurogenesis in HIV patients and in animal models of HIV neurotoxicity (gp120 tg and others) [13,36,37].

Although the physiological function of CRMP2 has been associated with neuronal development and neurite outgrowth [38,39], the effects of abnormal CDK5-directed CRMP2 hyperphosphorylation in adult neurogenesis have not been previously investigated. CRMP2 (also known as Dpysl2) is a signaling protein that has been shown to play a role in growth cone collapse and axon development [40]. It is a member of a group of related proteins including CRMP1 and CRMP4, all of which are highly abundant in the brain. CRMP2 has no known enzymatic activity [41], however its C-terminal region is highly Ser and Thr-rich, and is targeted for phosphorylation by a number of kinases, including CDK5, GSK3β [28,42], Rho kinase [43,44], and Fyn kinase [26], among others. Phosphorylation of CRMP2 has been reported for at least five amino acid residues in the C-terminal region, namely Thr509, Thr514, Ser518, Ser522 and Thr555. Phosphorylation by CDK5 at Ser522 acts as a priming site for subsequent phosphorylation by GSK3β at Thr514 [25,28], while other residues can be phosphorylated independently of CDK5 activity. It is possible that manipulation of phosphorylation at residues other than Ser522 of CRMP2 might also regulate neurite outgrowth, however because CDK5 is the only kinase that has been reported to phosphorylate the Ser522 residue, and since phosphorylation at this site acts a gatekeeper that regulates subsequent phosphorylation at other epitopes, we focused our studies on evaluating the specific role of phosphorylation at the Ser522 residue.

In support of a pivotal role for CDK5 in the phosphorylation of CRMP2, a previous study demonstrated that CRMP2 was not phosphorylated in *cdk5-/- *embryonic brain using an antibody that recognizes several phosphorylated epitopes of CRMP2 (Thr509, Ser518, and Ser522) [27]. Furthermore, the association of CRMP2 with tubulin was enhanced in *cdk5-/- *brains compared to wild-type brain, supporting the role of CDK5-mediated phosphorylation of CRMP2 in modulating CRMP2-tubulin interactions [27]. The functional effects of phosphorylation at the other Ser/Thr residues of CRMP2 is not entirely clear, however previous studies have shown that some of these post-translational modifications dramatically modulate protein-protein interactions between CRMP2 and its binding partners, including tubulin heterodimers [30]. Of interest, previous studies have also shown that CDK5 and p35 can associate with microtubules [45,46], and CDK5 substrates include a number of microtubule-associated proteins.

The results of the present study show that abnormal activation of CDK5 and subsequent hyperphosphorylation of CRMP2 may have detrimental effects on structural elements of developing neurons, such as microtubules and cytoskeletal organization. In support of this possibility, we showed that the alterations in neurite outgrowth and CRMP2 phosphorylation in p35-overexpressing NPC-derived neural progeny were accompanied by disrupted microtubule organization compared to controls. Live-cell imaging of polymerized tubulin, and ultrastructural analysis of microtubule structure in NPC-derived neural progeny revealed diffuse, mesh-like microtubule distribution under conditions of p35-mediated abnormal CDK5 activation. This was in stark contrast to more robust microtubules that were observed under normal differentiation conditions. Furthermore, control NPC-derived neural progeny displayed rapid re-polymerization of microtubules following chemical disruption with the microtubule-destabilization agent, nocodazole, while cultures expressing p35 showed only minimal re-polymerization even after further incubation post-washout. These observations are in line with previous studies suggesting that the mechanisms downstream of abnormal CDK5 activation may involve the destabilization of microtubules [47] and that CRMP2 plays a role in regulating microtubule dynamics [48]. Together, this supports a role for CDK5-mediated CRMP2 hyperphosphorylation in the mechanisms of defective adult neurogenesis in neurodegenerative conditions such as HIVE.

## Conclusions

Here we have shown that CRMP2 is a molecular mediator of abnormal CDK5 activation and impaired neuronal maturation in an *in vitro *model of adult neurogenesis. Abnormal activation of CDK5 by p35 overexpression, or under conditions of HIV protein-mediated toxicity, results in hyperphosphorylation of CRMP2 in an *in vitro *model of adult neurogenesis. The relevance of these results to disease mechanisms is supported by the observations of upregulated CRMP2 levels and phosphorylation in the brains of patients with HIVE and an *in vivo *model of HIV-gp120 mediated neurotoxicity. The microtubule abnormalities that accompanied CDK5 activation and CRMP2 hyperphosphorylation in the *in vitro *model support the possibility that alterations in p35/CDK5/CRMP2 signaling contribute to impaired neurogenesis by disturbing microtubule integrity in maturing neurons. Targeting the CDK5/CRMP2 pathway for therapeutic intervention might provide new approaches to ameliorate the neurogenic alterations associated with neurodegenerative conditions including HIVE.

## Methods

### Neuronal progenitor cell and primary neuronal cell culture and *in vitro *modeling of abnormal CDK5 activation in progenitor cell and mature neuron populations

Adult rat hippocampal (ARH) NPCs (Millipore, Temecula, CA) were cultured routinely for expansion essentially as previously described [49] with some modifications. Briefly, cells were grown for expansion in DMEM/F12 media (Mediatech, Manassas, VA) containing B27 supplement, 1X L-glutamine and 1X antibiotic-antimycotic (all from Invitrogen, Carlsbad, CA). For induction of neuronal differentiation, cells were plated onto poly-ornithine/laminin (Sigma-Aldrich, St. Louis, MO) coated plates or coverslips and transferred the next day to differentiation media containing N2 supplement (Invitrogen), 1 μM all-trans retinoic acid (Sigma-Aldrich), 5 μM forskolin (Sigma-Aldrich) and 1% FBS. Cells were differentiated for four days, and fresh differentiation media was added at day 2. It should be noted that this differentiation procedure generates heterogeneous cultures, and therefore we refer to the cells derived from the differentiation process as "NPC-derived neural progeny."

To induce CDK5/p35 activity *in vitro*, cells were infected on day 2 of differentiation with adenovirus expressing human p35 or GFP control (Vector Biolabs, Philadelphia, PA) at a multiplicity of infection (MOI) of 30. Additional control experiments were performed with cells transfected using BPfectin according to the manufacturer's guidelines (Biopioneer, San Diego, CA) with a plasmid expressing myc-tagged p35 (Addgene plasmid 1347, deposited by Dr. Li-Huei Tsai, Picower Institute, Cambridge, MA). Two days after infection or transfection with p35, cells were processed for immunoblot analysis with total cell lysates or immunocytochemical analysis with fixed cells on coverslips.

Similar experiments were performed in rat hippocampal primary neurons (ScienCell Research Laboratories, Carlsbad, CA) cultured on poly-lysine-coated plates and glass coverslips according to the supplier's recommendations in Neuronal Medium (ScienCell). For induction of CDK5/p35 activity, cells were infected two days after plating with p35-adv at an MOI of 100. A higher MOI was selected for the primary neuronal experiments because these cells were plated at a lower density than NPCs in culture. Two days after infection, cells were prepared for immunoblot or immunocytochemical analyses. A subset of experiments were performed with NPCs and primary hippocampal neurons where uninfected cells or cells infected p35-adv were exposed to recombinant gp120 protein for 24-48 hrs (100-200 ng/mL, LAV IIIB, Protein Sciences Corp., Meriden, CT) to model HIV protein associated neurotoxicity *in vitro*. At the conclusion of the *in vitro *experiments, cells were lysed for immunoblot analysis, or cells on coverslips were fixed in 4% paraformaldehyde (PFA) in phosphate-buffered saline (PBS) for immunocytochemical analysis.

### Site-directed mutagenesis and generation of hCRMP2 mutant constructs

The wild-type hCRMP2/pCMV6-XL4 plasmid DNA (Origene, Rockville, MD) was maintained in *E. coli *TOP10 cells (*dam*^+^/*dcm*^+ ^strain). Using plasmid DNA isolated from the *E. coli *strain as a template, site-directed mutagenesis of *hCRMP2 *was carried out using a QuickChange^® ^Lightning Site-Directed Mutagenesis Kit (Stratagene, La Jolla, CA) with the primers 5'-ctcggccaagacggctcctgccaagcag-3' (sense) and 5'-ctgcttggcaggagccgtcttggccgag-3' (antisense) for S522A single point mutation. The PCR reaction was performed according to the manufacturer's instructions, with 10-min extension cycles at 68°C. In order to remove the parental DNA template, PCR products were subjected to DpnI restriction enzyme digestion reaction and then directly transformed into *E. coli *TOP10 cells. After selection on LB medium supplemented with ampicillin, plasmid DNA was extracted from positive transformants using a QIAprep Spin Miniprep kit (Qiagen) according to the manufacturer's instructions. Purified plasmids were subjected to DNA sequencing with hCRMP2 specific primers: 5'-atcaaggcaaggagcaggct-3' (sense), and 5'-aatgttgtcatcaatctgagcacca-3' (antisense). After sequence confirmation, the pCMV6-XL4 vector constructs containing either the wild-type hCRMP2, or the S522A construct were used for transformation and amplification in XL10 *E. coli *cells (Stratagene).

### Cell culture treatments--Pharmacological treatments, and siRNA and CRMP2 plasmid DNA transfection

For inhibition of CDK5 activity, cells were treated at day 2 of differentiation with either the pharmacological CDK5 inhibitor Roscovitine (1-10 μM, Calbiochem, San Diego, CA) or transfected with siRNA against CDK5, CRMP2, or control non-targeting fluorescent-tagged siRNA (37.5-150 ng, 5 nM final concentration, Qiagen, Valencia, CA) using the HiPerfect transfection reagent (Qiagen) according to the manufacturer's protocol. For each target, at least two different siRNAs were tested, and the one with the highest efficacy was selected for subsequent experiments.

For overexpression of wild-type or mutant human (h)CRMP2 in NPCs, cells were differentiated from day 0 in medium without antibiotics, and transfected on day 2 of differentiation (6 hrs prior to virus infection) with pCMV6-XL4 plasmids hCRMP2-WT, or hCRMP2-S522A, or pCMV-GFP control. Transfection was performed using Lipofectamine 2000 transfection reagent (Invitrogen) at a concentration of 2.5 μL/mL according to the manufacturer's instructions. For transfection of cells in 6-well plates, 4.0 μg plasmid DNA was applied per well, and for transfection of cells on coverslips in 6 cm dishes, 8.0 μg plasmid DNA was applied per dish. Six hrs after transfection, fresh differentiation medium without antibiotics was applied with or without viral vectors. Cells were then lysed for biochemical analyses or fixed for immunocytochemical analysis. For disruption of microtubules, cells were treated with nocodazole (5 μg/mL, Sigma-Aldrich) for 3 hrs. Then, cultures were washed with differentiation media three times, followed by incubation in fresh media for 10 mins, 20 mins or 30 mins. Cells were then fixed with glutaraldehyde for tubulin immunofluorescence and neurite outgrowth analysis.

### Neurite outgrowth studies

A subset of cultured cells on coverslips were fixed in a solution containing glutaraldehyde, which better preserves the cytoskeleton for optimal visualization of neurites using immunofluorescence with an antibody against β-Tubulin. For this purpose, a fixation procedure was used essentially as previously described by Desai and Mitchison [50]. Briefly, media was gently aspirated from cells growing on glass coverslips, and then cells were extracted for 30 sec in cytoskeletal buffer (CB, 80 mM PIPES pH 6.8, 1 mM MgCl_2_, 4 mM EGTA) containing 0.5% freshly added Triton-X 100. Glutaraldehyde (Electron Microscopy Sciences, Hatfield, PA) was immediately added to the CB on the coverslips at a final concentration of 0.5%. Coverslips were incubated for 10 min at 37°C. Fixative was then removed and a freshly-prepared solution of 0.1% NaBH_4 _in PBS was added and samples were incubated for 7 min at room temperature to quench free glutaraldehyde. Coverslips were washed at least 3 times in PBS to remove the NaBH_4_, and samples were then processed for β-Tubulin immunofluorescence analysis.

For β-Tubulin immunofluorescence and neurite outgrowth studies, NPC-derived neural progeny growing on coverslips were fixed with glutaraldehyde and incubated with a mouse monoclonal primary anti-β-Tubulin antibody (1:250, Clone B2.1, Sigma-Aldrich) for 1 hr at room temperature and detected with FITC-conjugated secondary antibodies (1:75, Vector Laboratories). β-Tubulin-labeled coverslips were mounted under glass coverslips with ProLong Gold antifade reagent with DAPI (Invitrogen) and imaged with a fluorescent digital microscopy (Olympus). For analysis of neurite outgrowth of NPC-derived neural progeny immunolabeled with β-Tubulin, neurites were traced and lengths were measured using the ImageJ Program (NIH, Bethesda, MD) with the NeuronJ Plugin [51].

### Live-cell staining and imaging of polymerized tubulin in NPC-derived neural progeny

For staining of polymerized tubulin in live cells, NPC-derived neural progeny treated with vehicle control or p35-adv were grown on glass coverslips in 12-well plates as described above, and incubated with Tubulin Tracker Green (Invitrogen) according to the manufacturer's guidelines. Briefly, at day 4 of differentiation, media was removed from the cultures, and replaced with 1 mL of warm HBSS (containing Ca, Mg, and 4 mM HEPES buffer [HBSS/HEPES buffer]) with 150 nM Tubulin Tracker reagent. Plates were incubated for an additional 10 mins at 37°C, and then washed three times in warm HBSS/HEPES buffer. Coverslips were rapidly transferred to slides and imaged within 5 mins on a digital fluorescent Olympus microscope.

### Electron microscopy analysis

Briefly, as previously described [52], NPCs were plated in 35 mm dishes with a coverslip in the bottom (MatTek, Ashland, MA) and infected with p35-adv as described in the cell culture conditions. After 4 days of differentiation, cells were fixed in 1% glutaraldehyde in media, then fixed in osmium tetraoxide and embedded in epon araldite. Once the resin hardened, blocks with the cells were detached from the coverslips and mounted for sectioning with an ultramicrotome (Leica, Germany). Grids were analyzed with a Zeiss OM 10 electron microscope as previously described [53].

### HIV cases and neuropathological assessment

For the present study HIV+ cases with and without encephalitis were selected from a cohort of 43 HIV+ subjects from the HIV Neurobehavioral Research Center (HNRC) and California NeuroAIDS Tissue Network (CNTN) at the University of California, San Diego. Subjects were excluded in these analyses if they had a history of CNS opportunistic infections or non-HIV-related developmental, neurologic, psychiatric, or metabolic conditions that might affect CNS functioning (e.g., loss of consciousness exceeding 30 minutes, psychosis, substance dependence). A total of 16 age-matched cases were identified with and without encephalitis (n = 8 per group), and without other complications, for inclusion in the present study (Table 2). All cases had neuromedical and neuropsychological examinations within a median of 12 months before death. Most patients died as a result of acute bronchopneumonia or septicemia, and autopsy was performed within 24 hrs of death (Table 2). Autopsy findings were consistent with AIDS, and the associated pathology was most frequently due to systemic CMV, Kaposi sarcoma, and liver disease. In all cases, neuropathological assessment was performed in paraffin sections from the frontal, parietal, and temporal cortices, and the hippocampus, basal ganglia and brainstem stained with H&E, or immunolabeled with antibodies against p24 and glial fibrillary acidic protein (GFAP, marker of astrogliosis) [54,55]. The diagnosis of HIVE was based on the presence of microglial nodules, astrogliosis, HIV-p24 positive cells, and myelin pallor. Formalin-fixed sections and frozen brain samples were obtained from the hippocampus of HIV and HIVE cases for biochemical analysis. Brain tissue from HIV-infected subjects without evidence of neuroinflammation provides a close control for the systemic effects of HIV infection in the absence of neurodegenerative changes, and previous studies have shown that the CDK5 pathway is dysregulated specifically in cases with encephalitis compared to HIV-infected non-encephalitis cases [8,56]. For these reasons, and due to the scarcity of tissue samples available from age-matched non-HIV infected control subjects, HIV-positive cases without neuroinflammatory changes were used for comparison with cases with HIVE.

### Generation of GFAP-gp120 tg mice and crosses with CDK5-deficient mice

For studies of CDK5 activation in an animal model of HIV-protein mediated neurotoxicity, tg mice expressing high levels of gp120 under the control of the GFAP promoter were used [57]. These mice develop neurodegeneration accompanied by astrogliosis, microgliosis [57], and memory deficits in the water maze [58]. To study the effects of genetic CDK5 inhibition on CRMP2 expression and phosphorylation *in vivo*, CDK5 heterozygous-deficient mice (CDK5^+/-^) [59] were crossed with the GFAP-gp120 tg mice as previously described [8]. Full ablation of both copies of CDK5 (CDK5^-/-^) causes severe neurodevelopmental alterations, so in order to study CDK5 knockdown in the adult mouse brain, the CDK5^+/- ^animals were used as a model of reduced CDK5 activity. For *in vivo *studies, brain sections from 8-month old nontg, CDK5^+/-^, gp120 tg, or gp120 tg/CDK5^+/- ^crossed mice (n = 4 mice per group) were used for biochemical analysis of CRMP2 expression and phosphorylation.

### Tissue processing

In accordance with NIH guidelines for the humane treatment of animals, mice were anesthetized with chloral hydrate and flush-perfused transcardially with 0.9% saline. Brains were removed and divided sagittally. One hemibrain was post-fixed in phosphate-buffered 4% paraformaldehyde at 4°C for 48 hrs and sectioned at 40 μm with a Vibratome 2000, while the other hemibrain was snap frozen and stored at -70°C for protein analysis. All experiments described were approved by the animal subjects committee at the University of California at San Diego (UCSD) and were performed according to NIH recommendations for animal use.

### Immunoblot analysis

For immunoblot analysis, adherent cells in culture or mouse or human brain samples (0.1 g) were lysed in buffer composed of 10 mM Tris-HCl (pH 7.4), 150 mM NaCl, 5 mM EDTA (TNE) containing 1% Triton-X 100, homogenized using a microgrinder, and centrifuged at 10,000 rpm for 10 minutes to clear insoluble material. The supernatant was harvested, and protein content in the total cell lysates was determined using the bicinchoninic acid protein assay kit (Pierce, Rockford, IL). Then, 20 μg of each sample was separated by gel electrophoresis on 4-12% Bis-Tris gels (Invitrogen) and blotted onto 0.45 μm PVDF membranes (Millipore, Temecula, CA). All immunoblots were incubated in primary antibodies diluted in 5% BSA in PBS-Tween (PBS-T) overnight at 4°C. Immunoblots were probed with rabbit polyclonal antibodies against phosphorylated (pSer522) CRMP2 (1:1000, ECM Biosciences, Versailles, KY), phosphorylated (pThr514) CRMP2 (1:1000, Cell Signaling Technology, Danvers, MA), phosphorylated (pThr555) CRMP2 (1:1000, ECM Biosciences), total CRMP2 (1:1000, Millipore), p35/p25 (1:500, C-19, SantaCruz Biotechnology, Santa Cruz, CA), CDK5 (1:500, C-8, SantaCruz Biotechnology), or GFP (1:1000, Millipore); or mouse monoclonal antibodies against β-III Tubulin (1:5000, clone Tuj1, Covance), β-Tubulin (1:1000, clone B2.1, Sigma-Aldrich), or GFAP (1:1000, Millipore).

For immunoblot analysis with a panel of additional phosphorylated or total CRMP proteins, blots were probed with antibodies described in Table 1 at a dilution of 1:1000 for all antibodies. All immunoblots were stripped and probed with an antibody against actin (C4 clone, Millipore) as a loading control as previously described [53]. After incubation with primary antibodies, blots were incubated for 45 mins at room temperature in secondary antibodies diluted in 5% non-fat milk with 1% BSA in PBS-T. Blots were developed with enhanced chemiluminescence (Perkin-Elmer, Waltham, MA), and images were obtained and semi-quantitative analysis was performed using the VersaDoc gel imaging system and Quantity One software (Bio-Rad, Hercules, CA).

### Immunocytochemistry and image analysis

For immunocytochemical analysis, briefly, as previously described [60], cells on coverslips were fixed in 4% paraformaldehyde (PFA) in PBS and washed with Tris buffered saline (TBS, pH 7.4). For single-label immunostaining, coverslips were pre-treated in 3% H_2_O_2_, blocked with 10% serum (Vector Laboratories, Burlingame, CA), and incubated with a rabbit polyclonal primary antibody against phospho-CRMP2 (Ser522, 1:1500, ECM Biosciences) diluted in PBS-T, and detected with the Tyramide Signal Amplification™-Direct (Red) system (NEN Life Sciences, Boston, MA). Immunolabeled coverslips were mounted under glass coverslips with ProLong Gold antifade reagent with DAPI (Invitrogen) and imaged with a Zeiss 63X (N.A. 1.4) objective on an Axiovert 35 microscope (Zeiss, Germany) with an attached MRC1024 laser scanning confocal microscope system (BioRad) [61]. All samples were processed simultaneously under the same conditions and the experiments were performed twice to assess reproducibility. To confirm the specificity of primary antibodies, control experiments were performed where sections were incubated overnight in the absence of primary antibody (deleted) or primary antibody pre-incubated with blocking peptide.

For immunocytochemical analysis in human or mouse brain tissue, briefly as previously described [61] vibratome sections from the hippocampus (40 μm thick) of the HIV patients or from nontg, gp120 tg or CDK5^+/- ^mice were incubated with antibodies against phospho-CRMP2 (Ser522, 1:300, ECM Biosciences) or CRMP2 (1:300, Millipore). Primary antibody incubation was followed by incubation with secondary biotinylated IgG, then avidin-HRP and diaminobenzidine (DAB) detection as previously described [8]. Immunostained sections were imaged with a digital Olympus microscope and assessment of levels of immunoreactivity was performed utilizing the Image-Pro Plus program (Media Cybernetics, Silver Spring, MD).

For double-labeling analysis, coverslips or brain sections were incubated with a rabbit polyclonal primary antibody against phospho-CRMP2 (Ser522, 1:1500, ECM Biosciences) or CRMP2 (1:1500, Millipore) detected with Tyramide Red. The next day samples were co-labeled with mouse monoclonal antibodies against β-III Tubulin (1:250, Tuj1 clone, Covance) or MAP2 (1:100, Millipore), or a goat polyclonal antibody against the immature neuronal marker DCX (1:100, Santa Cruz Biotechnology), detected with FITC-conjugated secondary antibodies (1:75, Vector Laboratories). Samples were mounted and imaged as described above for single-labeling immunofluorescence analysis. For each sample a total of three sections (10 digital images per section at 400×) were analyzed in order to estimate the average number of immunolabeled cells per unit area (mm^2^) and the average intensity of the immunostaining (corrected optical density).

### Statistical Analysis

All experiments were performed blind coded and in triplicate. Values in the figures are expressed as means ± SEM. To determine the statistical significance, values were compared by unpaired two-tailed Student's t-test or by one-way ANOVA with post-hoc Dunnett's test when comparing differences to controls, or by one-way ANOVA with post-hoc Tukey-Kramer test when comparisons were made among groups. The differences were considered to be significant if p values were less than 0.05.

## List of abbreviations

AD: Alzheimer's disease; CDK5: cyclin-dependent kinase-5; HIVE: HIV encephalitis; NPCs: neural progenitor cells; SVZ: subventricular zone; DG: dentate gyrus; CRMP2: collapsin-response mediator protein-2; tg: transgenic; DCX: doublecortin; SGZ: subgranular zone; nontg: non-transgenic; PFA: paraformaldehyde; PBS: phosphate-buffered saline; HAND: HIV associated neurocognitive disorders

## Competing interests

The authors declare that they have no competing interests.

## Authors' contributions

LC participated in the conception, design and coordination of the study, carried out the *in vitro *experiments and biochemical assays, and drafted the manuscript. RR participated in the immunoblot studies, carried out the immunohistochemical studies in human and mouse brain sections, and provided critical review of the manuscript. CP carried out the immunocytochemical assays for *in vitro *experiments. WD provided human brain tissue samples and prepared tissue samples for immunoblot analysis. MTM carried out the electron microscopy studies. CA participated in the design of the study and provided critical review of the manuscript. ER carried out the breeding and crosses of transgenic mouse lines. EM conceived of the study, and participated in its design and coordination and helped to draft the manuscript. All authors read and approved the final manuscript.

## Supplementary Material

Additional file 1 **Supplementary Figures 1-3 and legends**. Three supplementary figures and the corresponding figure legends are provided as additional materials with the main manuscript submission. Click here for file
