# Supplementary material for: Phosphorylation of collapsin response mediator protein-2 disrupts neuronal maturation in a model of adult neurogenesis: Implications for neurodegenerative disorders
Source: Mol Neurodegener. 2011 Sep 24;6:67. doi: 10.1186/1750-1326-6-67 (PMC3204248; doi:10.1186/1750-1326-6-67)
Supplement: Additional file 1 — Supplementary Figures 1-3 and legends. Three supplementary figures and the corresponding figure legends are provided as additional materials with the main manuscript submission. [file 1750-1326-6-67-S1.PDF]

Supplemental figure legends

Figure S1 siRNA knockdown of CRMP2 abolishes hyperphosphorylation induced by abnormal CDK5 activation in NPC-derived neural progeny. Differentiating NPCs were transfected with siRNA against CRMP2 (siCRMP) at day two of differentiation, then infected with p35 adenovirus. Cell lysates were analyzed by immunoblot with antibodies against pSer522-CRMP2, tCRMP2, p35, and actin as a loading control. (A) siRNA knockdown of CRMP2 with two different siRNAs (siCRMP2 (#1) and siCRMP2 (#2)) reduced immunoreactivity with antibodies against total and phosphorylated (Ser522) CRMP2 under normal conditions and after exposure to p35 virus (box). (B,C) Semi-quantitative image analysis of immunoblots showing that treatment with siCRMP2 (#1) reduced total levels of CRMP2, and blocked p35/CDK5-mediated hyperphosphorylation at the Ser522 epitope. * $p < 0.05$ compared to vehicle-treated controls by one-way ANOVA with post-hoc Dunnett's test.

Figure S2 Site-directed mutagenesis and characterization of CRMP2 construct with a non-phosphorylatable CDK5 Ser522 epitope. A pCMV6-XL4 plasmid containing wild-type (WT) human CRMP2 was mutated at Ser522 to Ala (S522A-CRMP2) to prevent CDK5-mediated phosphorylation of this epitope. Differentiating NPCs were transfected on day 2 with plasmids containing WT or S522A-CRMP2, and on day 4, NPC-derived neural progeny were lysed for immunoblot analysis. (A) Diagram showing a portion of the Ser/Thr-rich C-terminal region of hCRMP2 where Ser522 was mutated to Ala. (B) Immunoblot analysis of lysates from NPC-derived neural progeny expressing WT-CRMP2, S522A-CRMP2, or CMV-GFP or transfection reagent (Lipofectamine, Lipo) controls. (C) Image analysis showing reduced pSer522-CRMP2 immunoreactivity in NPC-derived neural progeny expressing S522A-CRMP2 compared to WT-

CRMP2. Levels of total CRMP2 (tCRMP2) were similarly increased in cells expressing WT-CRMP2 and S522A-CRMP2 compared to vector-infected controls. * $p < 0.05$ compared to vehicle-treated controls by one-way ANOVA with post-hoc Dunnett's test. # $p < 0.05$ compared to p35-expressing NPCs by one-way ANOVA with post-hoc Tukey-Kramer test.

Figure S3 Increased CRMP2 phosphorylation in the brains of patients with HIV encephalitis. Total homogenates from the brains of HIV+ non-encephalitis and HIV encephalitis (HIVE) patients were processed for immunoblot analysis with antibodies against pSer522-CRMP2 and total (t)CRMP2. (A) CRMP2 phosphorylated at the CDK5 epitope (pSer522) was detected primarily as a single band at an approximate molecular weight of 64 kDa, and tCRMP2 was detected primarily as two bands at molecular weights of 62 and 64 kDa in HIV+ and HIVE patients. Actin was used as a loading control. (B) Semi-quantitative image analysis of pSer522-CRMP2 immunoreactivity by immunoblot in the brains of HIV+ patients. * $p < 0.05$ compared to HIV+ controls by unpaired two-tailed Student's t-test ($n = 8$ per group).

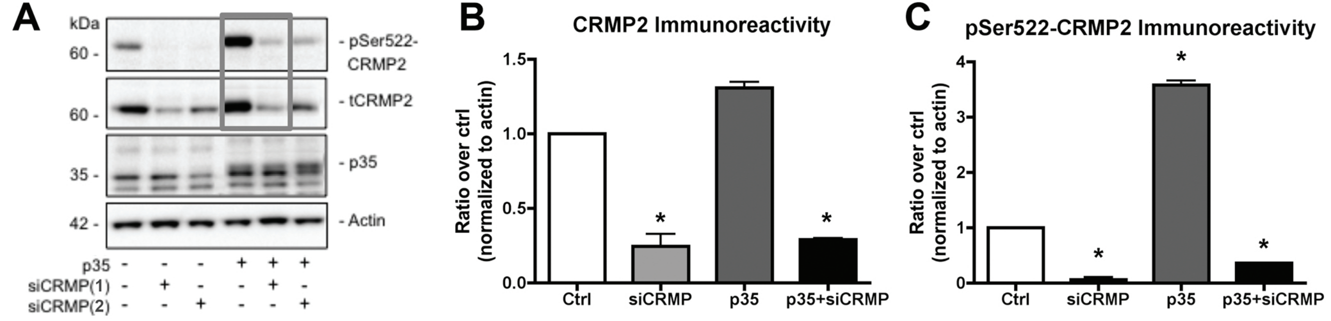
A
kDa
60 -
60 -
35 -
42 -
- pSer522-CRMP2
- tCRMP2
- p35
- Actin
p35 - - - + + +
siCRMP(1) - + - - + -
siCRMP(2) - - + - - +
B
CRMP2 Immunoreactivity
Ratio over ctrl (normalized to actin)
1.5
1.0
0.5
0.0
*
*
Ctrl
siCRMP
p35
p35+siCRMP
C
pSer522-CRMP2 Immunoreactivity
Ratio over ctrl (normalized to actin)
4
3
2
1
0
*
*
*
Ctrl
siCRMP
p35
p35+siCRMP

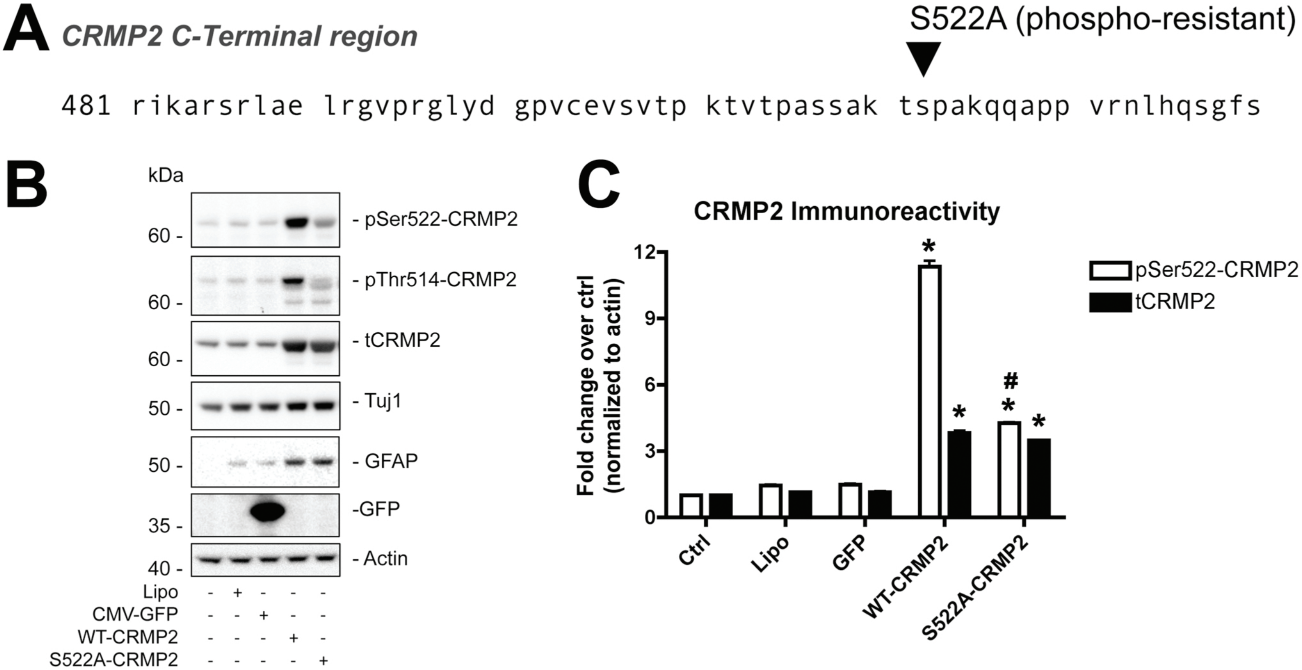
A
CRMP2 C-Terminal region
S522A (phospho-resistant)
481 rikarsrlae lrgvprglyd gpvcevsvtp ktvtpassak tspakqqapp vrnlhqsgfs
B
kDa
60 -
- pSer522-CRMP2
60 -
- pThr514-CRMP2
60 -
- tCRMP2
50 -
- Tuj1
50 -
- GFAP
35 -
-GFP
40 -
- Actin
Lipo - + - - -
CMV-GFP - - + - -
WT-CRMP2 - - - + -
S522A-CRMP2 - - - - +
C
CRMP2 Immunoreactivity
Fold change over ctrl (normalized to actin)
0
3
6
9
12
pSer522-CRMP2
tCRMP2
*
*
#
*
*
Ctrl
Lipo
GFP
WT-CRMP2
S522A-CRMP2

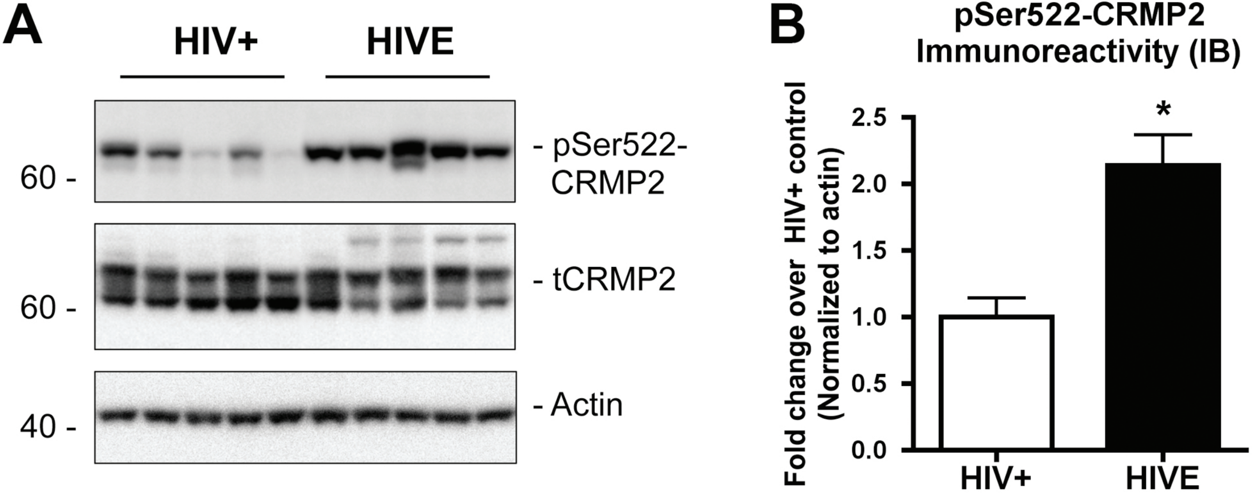
A
HIV+
HIVE
60 -
60 -
40 -
- pSer522-CRMP2
- tCRMP2
- Actin
B
pSer522-CRMP2 Immunoreactivity (IB)
Fold change over HIV+ control (Normalized to actin)
0.0
0.5
1.0
1.5
2.0
2.5
*
HIV+
HIVE
